# On a Robust, Sensitive Cell-Free Method for *Pseudomonas* Sensing and Quantification in Microfluidic Templated Hydrogels

**DOI:** 10.3390/mi10080506

**Published:** 2019-07-31

**Authors:** Jong Seto

**Affiliations:** 1 Department of Bioengineering and Therapeutic Sciences, University of California at San Francisco and California, Institute for Quantitative Biosciences (QB3), 1700 4th Street, Byers Hall #303, San Francisco, CA 94158, USA; JMSeto@lbl.gov; Tel.: +1-510-486-7343; 2 Molecular Foundry, Lawrence Berkeley National Laboratory, Berkeley, CA 94720, USA

**Keywords:** agarose, cell-free processes, quorum sensing, LasR, *Pseudomonas aeruginosa* infection

## Abstract

Through the use of droplet microfluidics to integrate cell-free activity into inert hydrogel beads, we have developed a platform that can perform biologically relevant functions without the need for cells. Specifically, cell-free lysates serve a utility in performing cellular functions and providing biologically relevant metabolic products without requiring the optimal biological conditions for cell growth and proliferation. By teasing out specific biological components that enable transcription and translation to occur, these cell-like functions can be reconstituted in vitro without requiring the entire cell and milieu of cellular organelles. This enables the optimization of synthetic biological circuits, either by concentration or logic switches, simply through the addition or removal of genetic components (plasmids, inducers, or repressors) of regulatory elements. Here, we demonstrate an application of cell-free processes that is robust and portable, independent of a substrate, to apply for sensing and reporting functions of a quorum-sensing molecule *N*-3-oxododecanoyl homoserine lactone (3OC12HSL) found crucial for pathological *Pseudomonas aeruginosa* infection. We develop an agarose bead platform that is easily adaptable and simply programmable to fit a variety of biological and chemical sensing applications for the utility of ease of delivery and activation in remote environments—even in conditions with very little hydration.

## 1. Introduction

Biological engineering of organismal and natural systems is ushering novel methods to examine disease and dysfunction in vivo [1,2,3,4,5]. Recent innovations using cell-free lysates have led to a variety of applications from bio-computation [6,7] molecular design and engineering [8,9], metabolic pathway engineering [10,11,12] to novel methods of protein synthesis [13,14,15]. In all these examples of cell-free lysate applications, the underlying premise of transcription and translation is still relied upon [16,17]. However, with modifications and the addition of synthetic energy pathways, these cellular processes can be engineered to operate ex vivo.

A range of cell types has been explored for cell-free lysates, most predominantly used for protein synthesis. These have included the use of lysates from ubiquitous *Escherichia coli* (*E. coli*) [6,18,19,20], rabbit reticulocytes, to wheat germ cells, all used for their specific properties. In the case of *E. coli*, the coupled transcription and translation machinery enable “one-pot” protein synthesis, but it is limited by the types of proteins synthesized such as post-translational modified proteins as well as mixed fraction proteins such as lipoproteins and proteins containing large portions of a carbohydrate moiety as found predominately in eukaryotic cells. While cell-free lysates from rabbit reticulocytes enable full length and post-translational modified proteins, protein yields are less desirable due to the presence of nucleases and other endogenous RNAs that inhibit efficient translation [14]. In comparison to wheat germ based cell-free lysates, exotic RNAs such as double-stranded RNAs and proteins with heavy thiol moieties are translated at high efficiencies, but these cell-free lysates require 5′ cap modifications to the mRNAs [15]. In examining the characteristics of each cell-free lysate in more detail under a variety of parameters, the function of these cell-free lysates can be further optimized.

Beyond uses for bulk protein synthesis, cell-free lysates are also the basis for precise input-output logic functions [5,21]. Several groups have explored the use of these components for diagnosis of disease and in detection of small molecules and environmental hazards [22]. Specifically, by combining the use of cell-free lysates to paper-based microfluidics, cell-free lysates provide the component of a detection device for the sensing of strain-specific pathogens like Ebola virus as well as toxins in the environment like arsenic [23,24,25]. However, due to the volume of samples needed to run paper-based cell-free diagnostics and the false positive detection rates, these devices may not be effective nor efficient in detecting pathogens and toxins in real-time. Limited volumes are especially pertinent when dealing with blood as an analyte. Others have improved the reliability and sensitivity of these devices using high-throughput microfluidics, but these also require the use of volumes >100 µL of analyte solutions [26,27]. One possible answer to the volume problem is to reduce the reaction volume and take advantage of the picomolar (0.001 nM) sensitivity of these lysates through the encapsulation of cell-free lysates in engineered small vessels that are semi-permeable to the environment.

In this study, we show a method utilizing de novo design combining the advantages of microfluidics techniques and biologically relevant quorum-sensing mechanisms to detect and report the presence of *Pseudomonas aeruginosa* infection into a single platform. This ability to contain and deliver the biosensor system in a cell-free compatible hydrogel improves the applicability and utility of cell-free processes in the field—with an emphasis on portability, sensitivity, and durability of these systems. Specifically, these cell-free hydrogels can be easily deployed in conditions where the utility of these systems would be much desired such as unfavorable biological and chemical environments. We demonstrate the successful utilization of co-localization of cell-free transcription and translation (TXTL) processes into a porous agarose microparticle for use as a biosensor and quantification tool.

## 2. Materials and Methods

### 2.1. Cell-Free Lysate and TXTL Extracts

Cell-free lysates were produced as described in Sun and coworkers [8,28]. The energy buffer and lysates were combined into a single tube before the encapsulation and lyophilization procedures. The LasR plasmid, GamS, and green fluorescence protein (GFP) constructs were gifts from the Laboratory of Richard M. Murray at Caltech.

### 2.2. Agarose Encapsulation of TXTL

Using a microfluidic flow-focusing device as seen in Abate et al. [29], an aqueous phase containing molten agarose (1%) along with the cell-free lysates, TXTL extracts, plasmids, and buffer were flowed into the device from one channel. The other channel fluorinated oil (3M 7500 HFE, St. Paul, MN, USA) containing 2% surfactant, as performed by Holtze et al. [30], were flowed to make monodisperse agarose beads which were ~40 µm in diameter.

### 2.3. Lyophilization and Storage

The agarose TXTL beads were collected immediately after the microfluidic generation of drops and allowed to equilibrate at room temperature for 1 h. Afterwards, the droplets were flash-frozen in liquid nitrogen and lyophilized overnight at a pressure of 30 mT (milliTorr) (Virtis Sentry 2.0, SP Scientific, Inc., Warminister, PA, USA). The resulting lyophilized agarose beads were kept in dry storage and under controlled humidity.

### 2.4. Fluorescence Microscopy

In order to image the GFP, an EVOS FL auto cell imaging microscope (Invitrogen EVOS FL microscope, Thermo Fisher Scientific, Inc., Waltham, MA, USA) with automated stage controller was used to capture GFP and Cy3 fluorescent images as well as the respective brightfield images.

### 2.5. RNA Analyses

Agarose TXTL beads were rehydrated and activated by the addition of 100 µL analytical grade ddH_2_O (reagent grade deionized water, Thermo Fisher Scientific, Inc., Waltham, MA, USA). The activated beads were subjected to RNA extraction via phenol: Chloroform treatment. The obtained RNA fraction was then prepared for analyses through the RNA 6000 Pico Chip Kit for activity and quality of the total RNA present in the samples. An Agilent 2100 bioanalyzer (2100 bioanalyzer, Agilent Technologies, Inc., Santa Clara, CA, USA) was used to quantify the RNA analyses.

### 2.6. Fluorescence Plate Reader

Plate reading measurements were conducted in 96-well flat, bottom well black plates (CellStar 96-well microplate, Grenier Bio-One GmbH, Kremsmuenster, Austria) with a multimode plate reader (M200 multimode plate reader, Tecan Group AG, Maennedorf, Switzerland) measurements from the top side. Overnight measurements were kept at constant 30 °C temperatures during the reads and shaken for 1 s before reads.

### 2.7. Fluorescence Activated Cell Sorting (FACS)

After activating and collecting the agarose TXTL beads, the samples were subjected to double emulsification by way of encapsulation with flow-focusing using a pluronic acid solution as the aqueous component such that the final double emulsified droplet size was ~50 µm in diameter. The double emulsified droplets were resuspended in water in a conical 5 mL test tube for mounting into the FACS (BD FACSAria, BD Biosciences, Inc., San Jose, CA, USA). Data was acquired and saved in the BD format. Further data analyses and extraction was performed with the FlowJo 10.0 (FlowJo, FlowJo LLC, Ashland, OR, USA) software suite.

### 2.8. 3D-Structure Illumination Microscopy (3D-SIM) and Wide-Field Deconvolution Fluorescence Microscopy

Both conventional fluorescence microscopy and three dimensional-structured illumination microscopy (3D-SIM) images were acquired using the DeltaVision OMX SR V4 system with Blaze SIM module (GE Healthcare Life Sciences, Chicago, IL, USA). The Deltavision OMX Blaze allows ultra-high-speed illumination and acquisition, as described previously [31].

Samples were prepared by adding a 20 µL drop of bead/bacteria mixture on a standard microscope slide and sandwiching between a 22 × 22 mm high precision coverslip (Bioscience Tools, San Diego, CA, USA). Images were captured with a 50 ms exposure and 5% laser power on a PCO Edge scientific CMOS cameras dedicated to the 488 nm channel using an Olympus PlanApo N 60× 1.42 NA oil objective and standard excitation and emission filter sets (in nm, 488 EX/500–550 EM) (Olympus America, Center Valley, PA, USA). 3D-SIM imaging was used to capture a small 4 or 6 µm thick sections of beads at a Z-spacing of every 125 nm. Unprocessed image stacks were composed of 15 images per z-section (five 72-degree phase-shifted images per angle at each of three interference pattern angles, +60, 0, and −60 degrees). Larger bead sections were acquired using the conventional widefield deconvolution imaging mode. 40 µm thick bead samples were captured using the same hardware as 3D-SIM experiments but at 1 um thick z-sections. Raw data from both 3D-SIM and widefield deconvolution modes were processed, deconvolved, and reconstructed in 3D using a constrained iterative algorithm (SoftWorX 4.0, GE Healthcare Life Sciences, Chicago, IL, USA). Reconstructed images were then exported and prepared for publication using ImageJ (NIH, Bethesda, MD, USA) and Avizo 3D software (FEI, Hillsboro, OR, USA) (Supplementary Materials Movie S1).

## 3. Results

**Figure 1:**
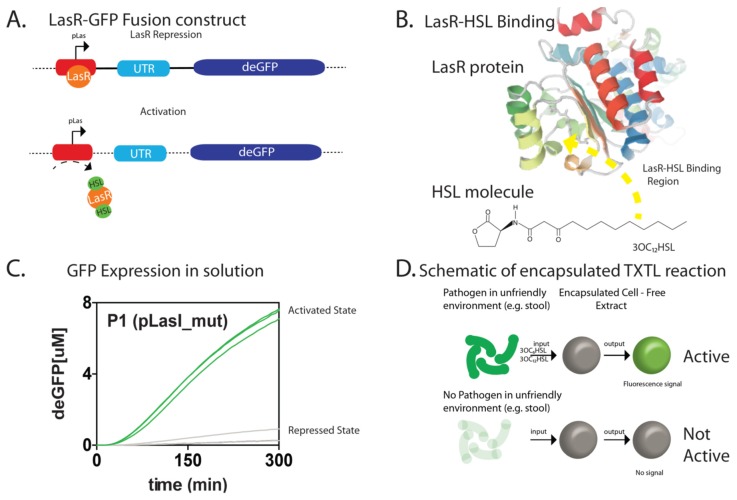
Properties of the LasR-based cell-free method in bulk and in agarose bead forms (**A**). A schematic of the interactions between homoserine lactones and LasR proteins in relation to LasR regulation of transcription (**B**). A green fluorescence protein (GFP) reporter system demonstrates the bulk regulation by LasR in transcriptional regulation (**C**). A crystal structure of the LasR-HSL bounded interaction (courtesy of PDB: 4Y90). (**D**) A schematic demonstrating the use of the LasR-based mechanism in agarose transcription and translation (TXTL) beads.

**Figure 2:**
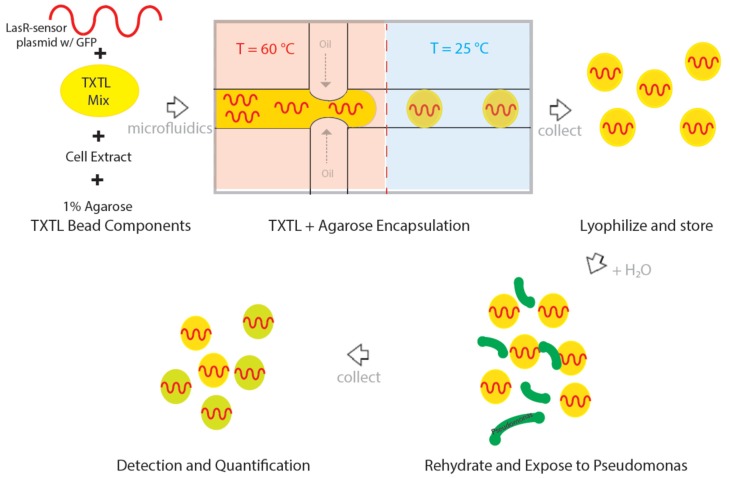
A schematic demonstrating the components of the LasR-based agarose TXTL bead formation and processing workflow for *Pseudomonas aeruginosa* sensing and reporting using microfluidic flow-focusing.

**Figure 3:**
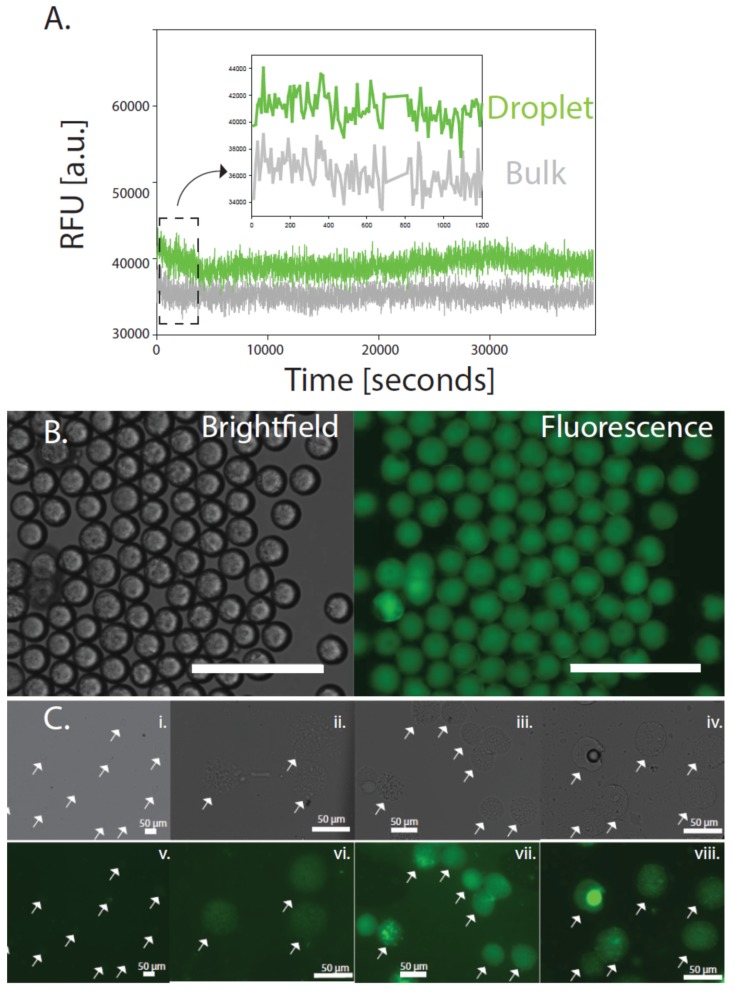
Agarose TXTL bead based sensing and quantification using the LasR-mechanism (**A**). The signal intensity differences observed between agarose bead versus bulk sensing (**B**). Brightfield and Fluorescence images of the agarose TXTL beads (scale bar = 1 mm) (**C**). Various concentrations of HSL (from 100 nM stock) added to the agarose beads to demonstrate HSL sensitivity in the LasR-based quorum-sensing system and subsequent fluorescence signal (**i**,**ii**,**iii**,**iv**) represent the brightfield image of the agarose beads and (**v**,**vi**,**vii**,**viii**) the corresponding fluorescent image, respectively. (**i**) 0 nM, (**ii**) 10 nM, (**iii**) 30 nM, (**iv**) 50 nM. The white arrows are showing the droplets under varying conditions of brightfield and fluorescence detection as a function of increased HSL (scale bar = 50 mm).

By coupling the transcription of the LasR repressor protein and a transcriptional lasR binding domain fused with a green fluorescent protein (GFP) expressing construct as the basis for sensing activated LasR states, a simple feedback loop utilizing the las quorum-sensing system can be construed as a form of a fluorescent marker within 30 min (Figure 1 and Figure 2). The specific binding region of the HSL molecule is based on the homodimerization into the binding pocket of the LasR protein whereby the N-terminal domain, the domain containing a sequence from 4 to 78 amino acids, binds and coordinates multimerization [32]. Upon binding and multimerization with HSL, the N- and C-termini are occupied and cannot participate in further transcriptional regulation. We utilize this binding event as a proxy for activation of the las quorum-sensing and in cells, this would activate virulence states. When coupled with an agarose shell, the cell-free TXTL results in a 20% improvement in fluorescence signal and prevents the nonspecific binding of contaminants (Figure 3A). The increased fluorescence is indeed a result of optimized cell-free TXTL reactions as confirmed through transcriptome analyses. By analyzing the rRNA in the beads compared to that in bulk solution, the amount of rRNA is dramatically lower in the functional agarose beads and the sensitivities are approximately four times higher in some cases (Supplementary Materials Figure S2).

Specifically, by simulating the activation of the las system through the presence of 3OC12HSL (HSL), this synthetic LasR sensing and reporter system can be tested as a function of various concentrations in vitro. Using a 100 nM concentration of HSL, we turn “on” the LasR-GFP and enable it to produce GFP. The homogenous fluorescence produced indicates that this concentration is saturation for the las system and indeed required for multimerization as supported by other groups [32]. As shown in Figure 3B, the 40 µm agarose TXTL beads fluoresce homogeneously throughout. At lower concentrations of HSL, the agarose TXTL beads fluorescence signal is tempered and is still visible even at 10 nM concentrations of HSL.

**Figure 4:**
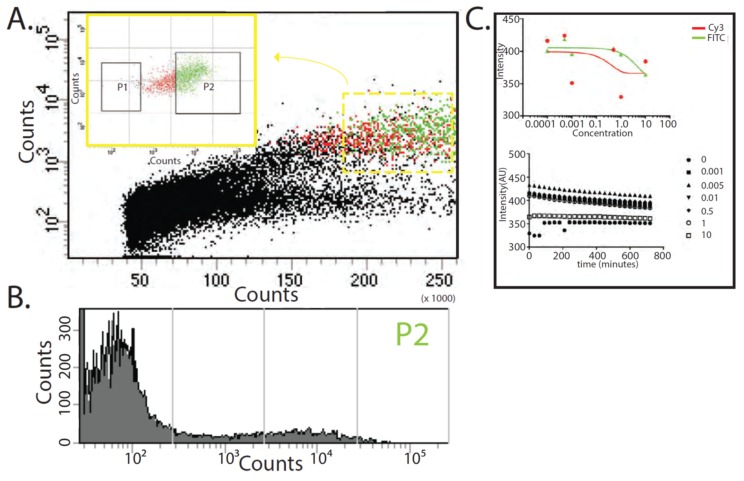
Quantifying the characteristics of the agarose TXTL bead in vitro (**A**). Fluorescence-activated cell sorting of the agarose TXTL bead sensing system with Cy5 as a background marker (**B**). Separating out the false positives, the actual GFP signal can be quantified such that we find that approximately 10% of the total number of beads measured (**C**). Dose independent behavior indicates the sensitivity of the agarose TXTL bead is closer to the nM than the µM concentrations of HSL as well as the effects of these doses over time confirming stability of the sensor.

To examine the fluorescence activity of the agarose TXTL beads over a population of beads, we found through fluorescence-activated cell sorting (FACS) as a method able to quantify the cell-like behaviors with necessary single agarose bead accuracy as well as the total number of beads that are activated. We can deliberately differentiate false-positive populations from genuinely active fluorescent signals intentionally introducing a Cy3 (red) background signal into all agarose TXTL beads. This provides the ability to differentiate populations which may be aberrant in behavior i.e., those beads that fluorescence green should also fluorescence read. We observe that approximately 1 in 10 fluorescent beads are indeed active and produce GFP with the correct Cy3 background (Figure 4A and Supplementary Materials Figure S4). This validates the total activity of the population of active beads.

Interestingly, when examining the effective concentrations of HSL used in vitro from 10 nM to 10 µM concentrations with the agarose TXTL beads as a ligand, the ligand-HSL interaction should follow a classical dose-response curve. However, in attempts to determine this dose-response curve of HSL and the agarose TXTL bead system, a non-sigmoid curve is observed (Figure 4B). This suggests the conditions for a standard dose-response curve do not exist, specifically the concentrations of the ligand-analyte behavior are at lower concentrations than those tested here. This behavior does not change tremendously with an increase of concentration in the ligand, the agarose TXTL beads (Supplementary Materials Figure S3A).

Additionally, another characteristic of fluorescence behavior is the lifetime of the signal. In evaluating the lifetimes of the fluorescent activity, the agarose TXTL system can produce a signal that will be active for over 12 h (Figure 4B and Supplementary Materials Figure S3B). Since the LasR mechanism created to make GFP does not include a degradation process of GFP, the signal produced is static and will only attenuate due to light attenuation from photobleaching. The negative slope of GFP produced from HSL concentrations between 0 and 10 µM is, in fact, a result of the bleaching from measurements and can be measured as a slope of −0.7 (Figure 4B). To ameliorate this effect, other modes of detection can be utilized and may, in fact, be more accurate in accessing the state of the las quorum-sensing system without observational artifacts such as through colorimetric and small molecule routes.

**Figure 5:**
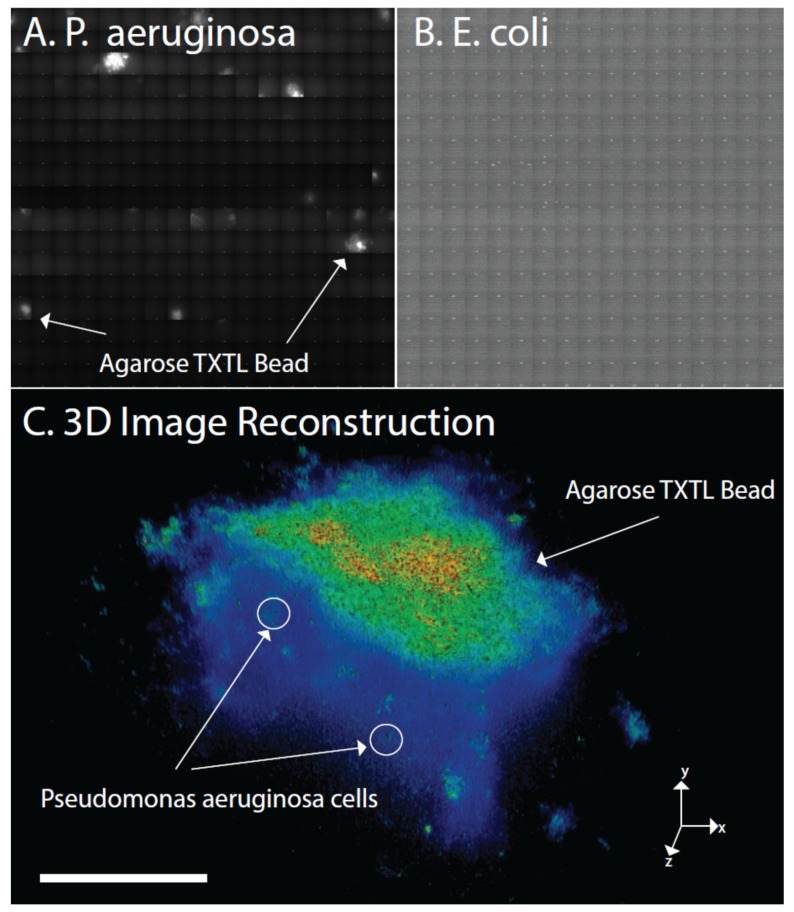
Imaging the sensing and reporting activities of agarose TXTL beads in cell cultures of (**A**), *Pseudomonas aeruginosa* (**B**), *Escherichia coli* control (**C**), 3D Reconstructed image of individual *Pseudomonas aeruginosa* cells and agarose TXTL bead (scale bar = 30 µm).

To validate that the LasR-GFP biosensor is indeed not producing false positives, we measure the utility of this system in cell cultures that do use the las system (Figure 5A) and those not using the las quorum-sensing system (Figure 5B). Specifically, by growing *Pseudomonas aeruginosa* (*P. aeruginosa*) cells in culture a positive control can be used to detect whether the agarose TXTL system is indeed functional. Whereas in the opposite scenario, by using *E. coli*, a cell which does not use the las quorum-sensing system, a negative control for the use of agarose TXTL system in vitro can be assessed. As shown in Figure 5, a culture of *P. aeruginosa* and *E. coli* cells is grown for 12 h in agar plate with the appropriate media and swabbed onto glass objective slides containing agarose TXTL beads. Using a 3D-SIM microscope, we raster the respective slide for 30 min to obtain sub-micron resolution over a large wide field. We find that the agarose TXTL beads indeed only functions with *Pseudomonas aeruginosa* cells.

## 4. Discussion

In the engineering of a cell-free lysate system coupled with a LasR-based transcriptional activation mechanism, a model system for the detection of quorum-sensing molecules can be utilized for prototyping of a biosensor. Specifically, several species such as *Salmonella typhimurium*, *Vibrio harveyi*, as well as *Pseudomonas aeruginosa* utilize the las system for regulation of cell density, where endogenous LasR repressor protein is induced by homoserine lactones (HSL) to activate the transcriptional activities for proteases and other enzymatic processes that inhibit cellular proliferation [33,34]. The presence of HSL molecules enables LasR to form multimer assemblies such that LasR is unable to bind to the promoter regions of genes that encode for these proteases [35,36]. Additionally, only through suppression of LasR are the genes responsible for virulence, cellular growth and development activated in vivo. In isolating the sensing component from the quorum-sensing complex and coupling a reporter, a benign LasR-based biosensor having no virulent properties can be constructed for detection and reporting of *Pseudomonas* growth (Figure 1). Specifically, a lasR-based inducible promoter coupled to a GFP construct is utilized to fluoresce when a critical threshold of an acyl-HSL, specifically *N*-3-oxododecanoyl homoserine lactone (3OC12HSL), is present. Under *Pseudomonas aeruginosa* (*P. aeruginosa*) virulence, HSL causes the LasR protein to unbind from the promoter and allows expression of virulent genes. In this case, expression of a GFP reporter is activated and “virulent states” can be observed optically (Figure 1 and Figure 3). Here, a LasR-based inducible transcription mechanism typically relied upon for regulation of virulence genes responsible for DNA replication, RNA transcription and translation, biofilm formation, and antibiotic resistance is co-opted for use in translation of a GFP reporter in the sensing and reporting of *Pseudomonas* infection.

In integrating this LasR-based mechanism into a delivery system that is permeable, size-controlled and chemically inert, the reaction volumes required for efficient detection is decreased and sensitivity range is increased by being able to tune the effective concentrations of the components that make up the system unlike other groups [20,37]. This delivery system is based on the use of hydrogel agarose microscale beads which we utilize to encapsulate the cell-free lysate and couple with transcription and translation (TXTL) processes (Figure 2). The size of the final microscale beads can be adjusted from diameters of 10–100 µm with minor adjustments to flow and pressure to the microfluidic processing and the porosity is an innate property of the cross-linking density of the agarose which can be controlled by agarose concentration [38,39]. Unlike other findings, the use of agarose is not toxic for the LasR quorum-sensing mechanism [28,40]. The resulting cell-free hydrogel device is a robust biosensor that can be delivered to both biologically and chemically hostile environments for controlled sensing and signaling of *P. aeruginosa* infection.

The formation of the agarose beads encapsulating the TXTL LasR process is performed through a controlled flow-focusing microfluidic device using fluorinated oils and aqueous phase [41,42] (Figure 2). The controlled flow of an aqueous component into an oil phase allows for the precise formation of aqueous droplets encapsulated by oil through phase separation (Supplementary Materials Figure S1). The low concentration of agarose utilized (1%) enables achieving a molten phase at 45 °C, a temperature that does not appear to degrade or denature the LasR protein-based mechanism of detection. The 1:1 agarose:aqueous phase (consisting of the TXTL components in water) is contained in a microfluidic droplet and allowed to cool such that the agarose phase transitions from a molten phase to a solid phase incorporating the TXTL components in its porous structure (Figure 2 and Supplementary Materials Figure S1). These TXTL extracts have been shown previously to be fully functional after lyophilization and reconstitution processes [37]. Accordingly, these agarose TXTL beads undergo further a lyophilization process to prevent diffusion of TXTL components from the bulk of the agarose bead to the surface to enable long term storage.

By hydrating the lyophilized agarose TXTL beads, the LasR-based detection mechanism is activated and ready to signal the presence of any HSL in solution. This signal is itself roughly 20% more intense than the same LasR signaling mechanism performed in a bulk solution and can last even longer (Figure 3 and Figure 4) using the same volumes. As reported in previous literature, the GFP signal is indeed HSL induced and a function of the LasR protein concentration found in the TXTL reaction [43,44]. This is also the case of the agarose TXTL beads where at approximately 3 µL of the stock lasR plasmid (100 nM lasR stock) used was found to provide for a stochastic signal of the GFP, indicating that at this concentration of LasR, there is a Poisson’s distribution of binding with LasR and HSL, and subsequently translation of the GFP signal (Supplementary Materials Figure S4A). This distribution is also validated by fluorescence-activated cell sorting (FACS) of the fluorescent beads which observes two distinct populations (approximately 10% are fluorescent of the total number of beads measured) of fluorescent agarose TXTL beads in the same concentration range of 1 µM HSL (Figure 4A,B and Supplementary Materials Figure S4). Beyond this concentration of LasR utilized in the TXTL reaction, a shift of an oversupply of LasR binding is found. This is noteworthy since LasR binding to HSL should be highly dependent on competitive binding due to the multimeric binding of LasR to HSL, further indicating that no cooperativity exists in the LasR-HSL interaction. It may be indicative of an assembly mechanism with LasR such that a cooperative binding mechanism may occur whereby multiple LasR are bound to a single molecule of HSL [32,34]. This would explain the shift in GFP production as well as LasR binding to HSL even in excess of LasR molecules (Supplementary Materials Figure S3A).

A caveat to utilizing a construct based on T7 RNA polymerase and linear plasmid system to reconstitute the LasR-based mechanism in TXTL is the innate degradation mechanisms in *E. coli* that serve to prevent foreign genomic material as well as errors in transcripts from propagating into the cell cycle through the activity of endo- and exo-nucleases [45,46]. In order to overcome these natural mechanisms inherent in *E. coli* based TXTL extracts that degrade exogenous oligonucleotides, nuclease inhibitors are utilized. We observe that with the addition of GamS, a small molecule inhibitor of exonuclease activity inherent in these TXTL extracts [8,20], the LasR mechanism is observed to possess increased signal sensitivity. The addition of a GamS component to the aqueous fraction during the formation of the agarose TXTL beads results in a 30% increase of signal observed immediately in the sensing and reporting of the presence of HSL using the LasR mechanism in comparison to the same population of agarose TXTL beads without GamS treatment (Supplementary Materials Figure S3B). Over a course of 12 h, both the non-GamS and GamS treated agarose TXTL beads reach a plateau in signal (Supplementary Materials Figure S3). This indicates that exonuclease activity is time-dependent, and activity is concentrated in the initial rounds of transcription and translation (Supplementary Materials Figure S3B). The activity of exonucleases in the absence of nuclease inhibitors like GamS can be suppressed by using excess exogenous plasmids such that the transcription and translation activities run longer than the exonuclease activity.

Contrary to standard concentrations utilized to evaluate dose-dependency of LasR quorum-sensing in bulk, the concentrations utilized here in the agarose TXTL beads using similar fluorescence imaging are in the saturation range where the doses result in dose-independent responses (Figure 4C). This indicates that the regime of the dose dependence is lower from the concentration of the lowest concentration range utilized here,10 nM, implicating the quorum-sensing of the LasR system in the form of agarose TXTL beads is on the order of pM sensitivity. In fact, by increasing the concentration 4 orders of magnitude higher to 10 µM, to that observed in the bulk TXTL reactions, there is a further decrease in fluorescence indicating dose independent behavior and suggesting the concentrations are too high for the LasR-HSL interactions (Figure 4C).

To evaluate the compatibility of this agarose TXTL bead platform for *Pseudomonas aeruginosa* detection in vivo, high spatial and temporal resolution obtained from 3D-structurured illumination microscopy (3D-SIM) is utilized to track the spatial interactions of this system in the presence of live cells [31]. Traditionally, one difficulty to perform such observations is the ability to obtain enough depth of field to accurately obtain a representative cell population as well as high sensitivity to resolve and differentiate individual cells in the sample. This 3D-SIM method enables the imaging of both the agarose TXTL bead as well as features found in single micron scale *Pseudomonas aeruginosa* cells (Figure 5). Specifically, a *Pseudomonas aeruginosa* strain (ATCC # BAA-1744) is initially incubated to obtain the mid-log phase of cell growth and is exposed to the agarose TXTL bead which results in the fluorescence of the bead. The media used for the mid-log phase of the cell cultures is also tested to determine whether the beads demonstrate fluorescence without cells. As shown in Figure 5, the *Pseudomonas aeruginosa* cell culture contains the autoinducer and triggers fluorescence in the beads. In previous plate-based experiments utilizing *Pseudomonas aeruginosa* cultures found in fecal tissue, the agarose TXTL beads were able to report induced signals (Supplementary Materials Figure S5). Additionally, the induced fluorescence demonstrates that the nM concentration of LasR protein produced from live cells is indeed within the thresholds of the agarose TXTL bead platform for rapid and detection of *Pseudomonas aeruginosa* in vivo as well.

Further modification of the agarose TXTL system to optimize detection of not only *Pseudomonas aeruginosa* infections, but also other quorum-sensing based infections in general is shown here as feasible. As shown in Supplementary Materials Figure S6, the LasR quorum-sensing is conserved within several bacterial groups and subsequently, the agarose TXTL bead platform would be effective in detecting the infections of other respective bacteria [47]. The binding of LasR to the respective DNA motifs can be analyzed through DNA footprint analyses [48]. As shown in Supplementary Materials Figure S6B, the DNA motifs utilized by various bacteria are themselves conserved, while maintaining a high affinity for LasR. This indicates that bacteria have co-evolved to utilize LasR transcriptional activities. More realistically, in cases of infection involving multiple bacteria, the agarose TXTL bead system itself can be modified so that multiple quorum-sensing mechanisms representing a diversity of bacteria can be embedded enabling the detection of multiple infections to be scaled-up appropriately.

Here, we have demonstrated the ability to design a biosensor-reporter pair integrated into agarose TXTL beads based on genetic circuits with a known input-output relationship, but it is also possible to leverage the high-throughput scalability of droplet-based microfluidics to identify gene circuits that detect novel input signals and optimize reaction conditions. As detailed in previous reports, libraries containing millions of unique gene combinations can be assembled in discrete agarose TXTL beads and assayed for reporter activity [49,50]. Agarose TXTL beads containing sensitive and specific sensor-reporter circuits can be enriched by FACS-like sorting procedure and the DNA contents can be sequenced to identify the constituent genes [51]. Although this approach does not require foreknowledge of endogenous regulatory responses, the efficiency of engineering detectors may be improved by enabling gene libraries to be screened on published transcriptomic data describing gene expression responses to specific signal inputs. We anticipate that these approaches will lead to the development of sensors for a broad range of molecular inputs such as bacterial stress signals and virulence factors which could provide important information related to mechanisms of antibiotic resistance and biofilm formation occurring in the analyte [52,53,54].

## 5. Conclusions

In this work, the utility of a cell-free agarose TXTL bead platform for rapid detection and sensing of *Pseudomonas aeruginosa* infections is shown. By having early detection and quick quantification of *Pseudomonas aeruginosa* specific infection, diagnosis and treatment to address the initial stages of pathogenesis can assist in limiting the duration and severity of illness as well as excessive use of antibiotic drugs. The agarose TXTL bead platform can also accommodate genetic reporter circuits that detect the presence of pathogens other than Pseudomonas as well as small molecules that carry additional information such as bacterial signals promoting virulence or antibiotic resistance [52,53,54,55]. As this platform is amenable to multiplexing of sensors, it will be possible to assay several parameters simultaneously for rapid assessment of pathogen prevalence and population state using different routes of detection (i.e., colorimetric, small molecular dyes such as TRITC, calcein, Cascade Blue) [56]. As shown in this work, unlike other in-vivo-engineered detection systems of pathogens [44], this platform can be stored in dry and hostile environments indefinitely and deployed within minutes with very little hydration <100 µL. From the thorough understanding of the components of this integrated agarose TXTL bead platform, where each component can be further engineered and optimized, the mechanisms involved can be configured according to very specific applications (Supplementary Materials Figure S6C). Through the use of directed evolution, each of these TXTL components can also be optimized through iteration for each application to ensure improved sensing and reporting [57,58]. The ease in adoption to a multitude of sensing applications along with its simple programmability and deployment makes the use of cell-free agarose TXTL bead platforms ideal and promising for a diverse range of specific biological and chemical sensing and detection tasks.

## Supplementary Materials

The following are available online at https://www.mdpi.com/2072-666X/10/8/506/s1, Figure S1: Interferometric imaging of the emulsions containing agarose TXTL beads (A) Bright field imaging [scale bar = 50 microns]; (B) Tomographic reconstruction of the respective TXTL beads found in panel (A) [scale bar = 50 microns] From the refractive differences of the emulsions containing agarose beads, the various colors indicate different phases, the utilized components from the oil-surfactant outer layer of the emulsion (green) to the agarose beads themselves (white). Figure S2: Total RNA sequence analyses of bulk and agarose TXTL bead using the same LasR-based quorum sensing and reporting mechanism with different concentrations of LasR in each respective scenario. Figure S3: Stabilization of the LasR-based GFP signal (A) By varying the LasR linear plasmid concentration, the GFP signal can be modulated [scale bar = 100 microns]; (B) The effect of the GFP signal following the addition of GamS to protect the linear plasmid from nucleases, in comparison to a non-GamS sample, can be observed to improve the GFP signal instantaneously [scale bar = 100 microns]. Figure S4: Flow cytometry/FACS of the fractions of the agarose TXTL beads activated with HSL and fluorescing (A) The total population sorted by flow cytometry and gated appropriately; (B) The activated population (green) differentiated by the non-activated population (red). Figure S5: Plot of GFP signal intensity of the LasR-based agarose TXTL bead biosensor system in a real-world biological environment such as fecal analyte observed via plate-based fluorescence assay as a function of concentration of analyte. Figure S6: Sequence analyses of LasR and LasR-like homologs (A) At the protein sequence level to demonstrate relationships to other transcriptional activators; (B) Of the interacting transcriptional binding footprints of LasR-like proteins found in various species; (C) A schematic demonstrating the possible concept of adapting agarose TXTL beads for diverse applications [scale bar = 100 microns]. Movie S1: 3D SIM imaging of the agarose TXTL beads in culture with *Pseudomonas* cells. An animation of the 3D reconstruction of *Pseudomonas aeruginosa* and activated agarose TXTL beads.
