# Supplementary material for: On a Robust, Sensitive Cell-Free Method for Pseudomonas Sensing and Quantification in Microfluidic Templated Hydrogels
Source: Micromachines (Basel). 2019 Jul 31;10(8):506. doi: 10.3390/mi10080506 (PMC6723077; doi:10.3390/mi10080506)
Supplement: Supplementary file 1 [file micromachines-10-00506-s001.zip › micromachines-531002-supplementary figures.pdf]

Figure S1

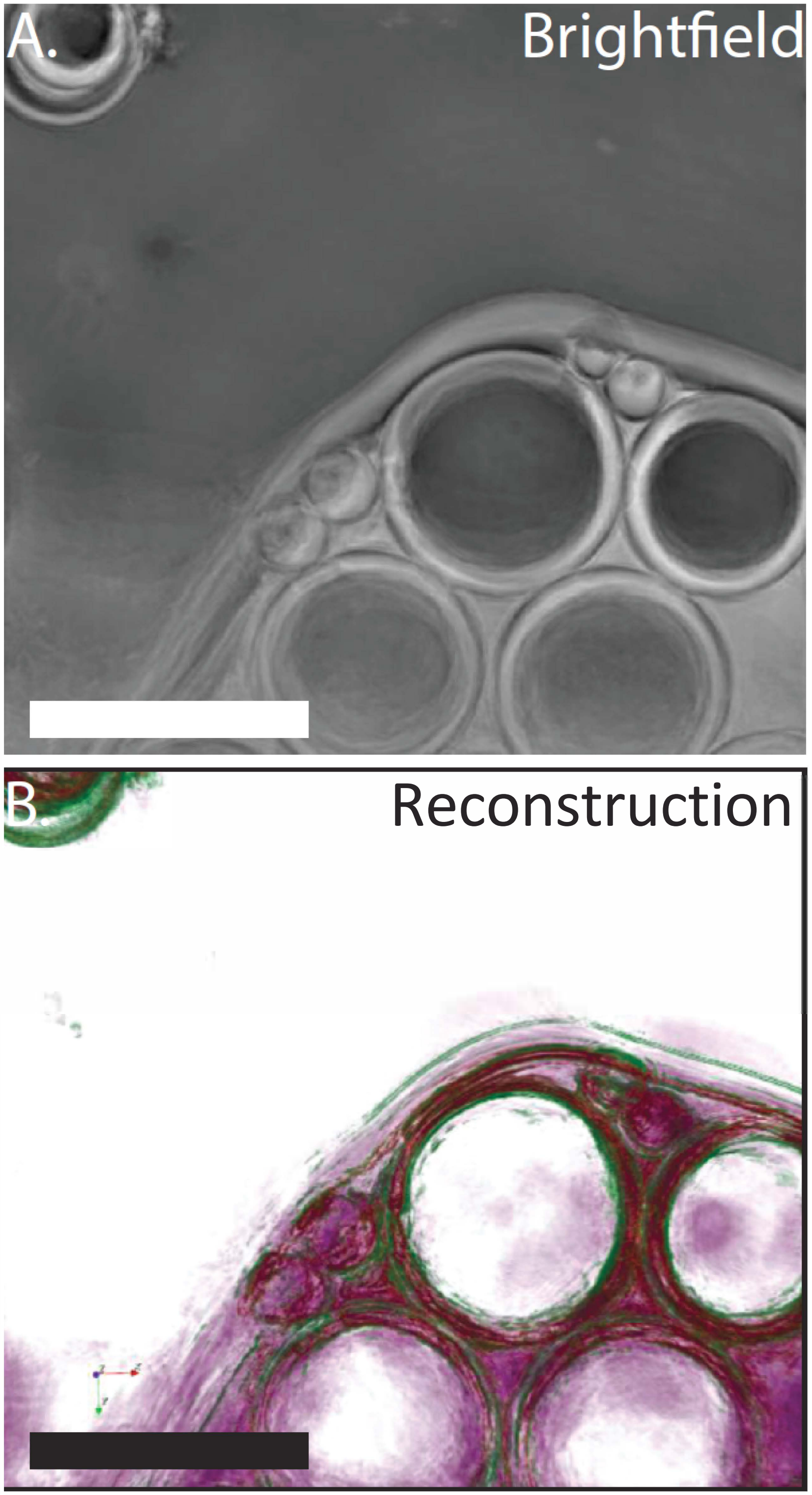

Figure S1. Interferometric imaging of the emulsions containing agarose TXTL beads (A.) Bright field imaging [scale bar = 50 microns] (B.) Tomographic reconstruction of the respective TXTL beads found in panel (A.) [scale bar = 50 microns] From the refractive differences of the emulsions containing agarose beads, the various colors indicate different phases, the utilized components from the oil-surfactant outer layer of the emulsion (green) to the agarose beads themselves (white).

Figure S2

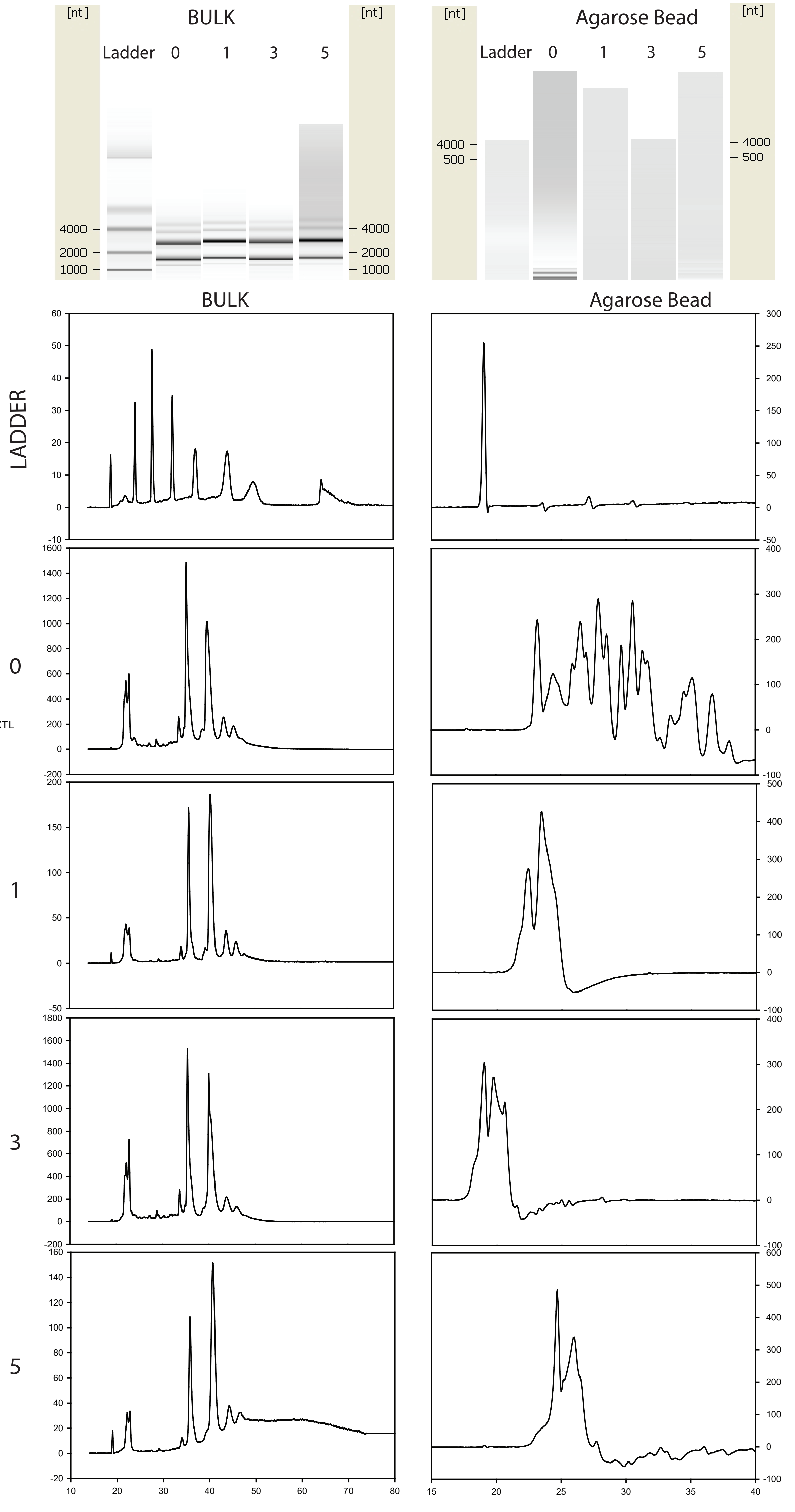

Figure S2. Total RNA sequence analyses of bulk and agarose TXTL bead using the same LasR-based quorum sensing and reporting mechanism with different concentrations of LasR in each respective scenario

Figure S3

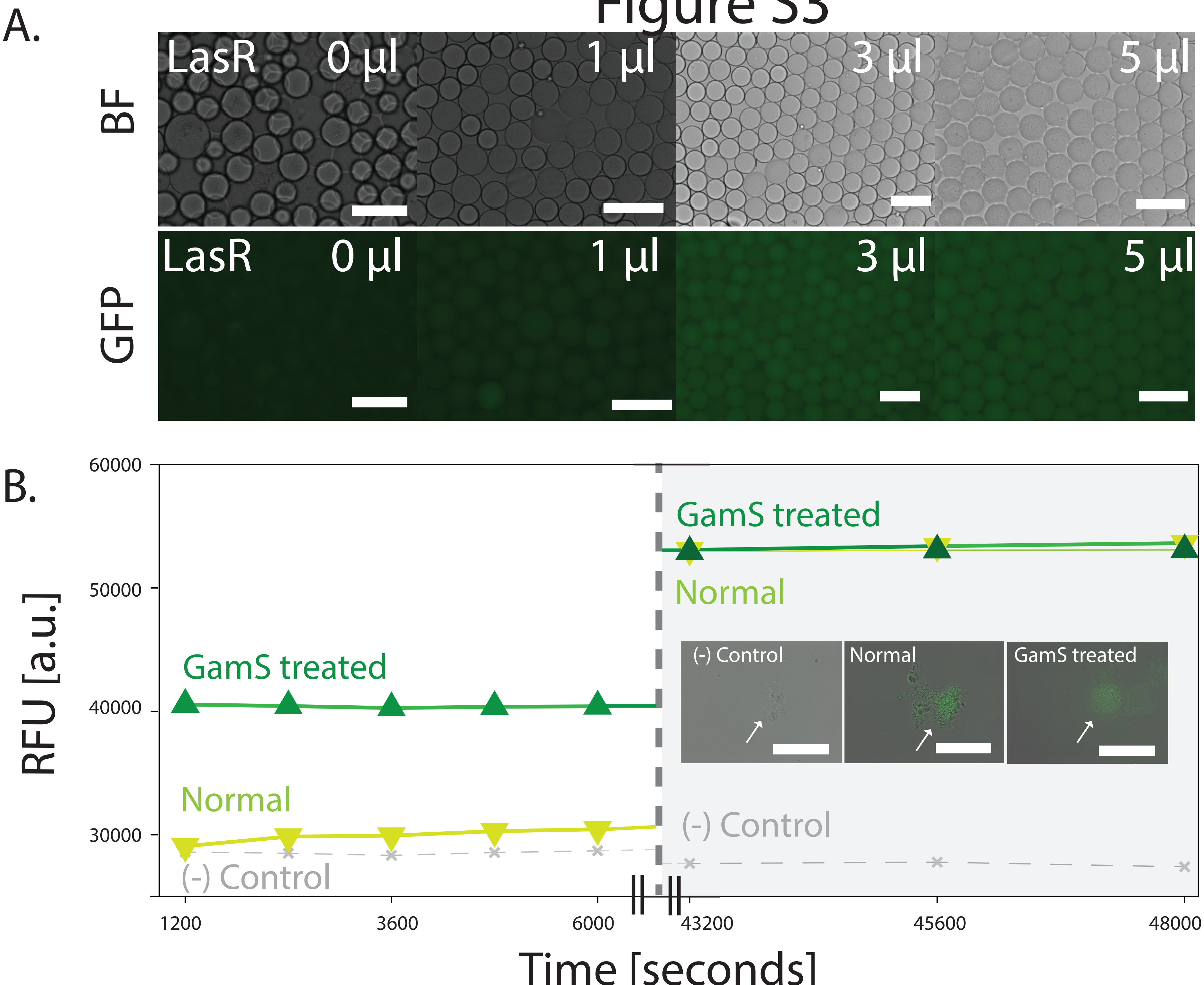

Figure S3. Stabilization of the LasR-based GFP signal (A.) By varying the LasR linear plasmid concentration, the GFP signal can be modulated [scale bar = 100 microns] (B.) The effect of the GFP signal following the addition of GamS to protect the linear plasmid from nucleases, in comparison to a non-GamS sample, can be observed to improve the GFP signal instantaneously [scale bar = 100 microns].

Figure S4

A.

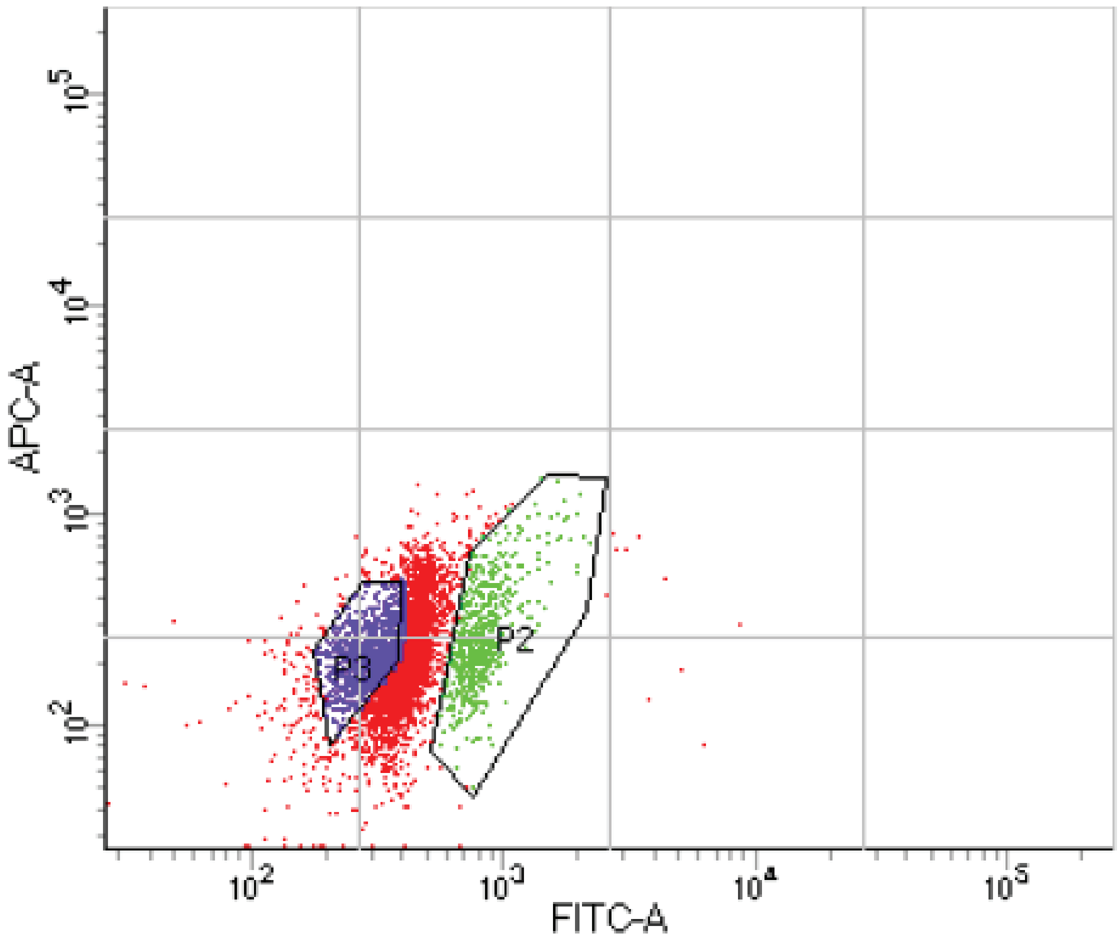

B.

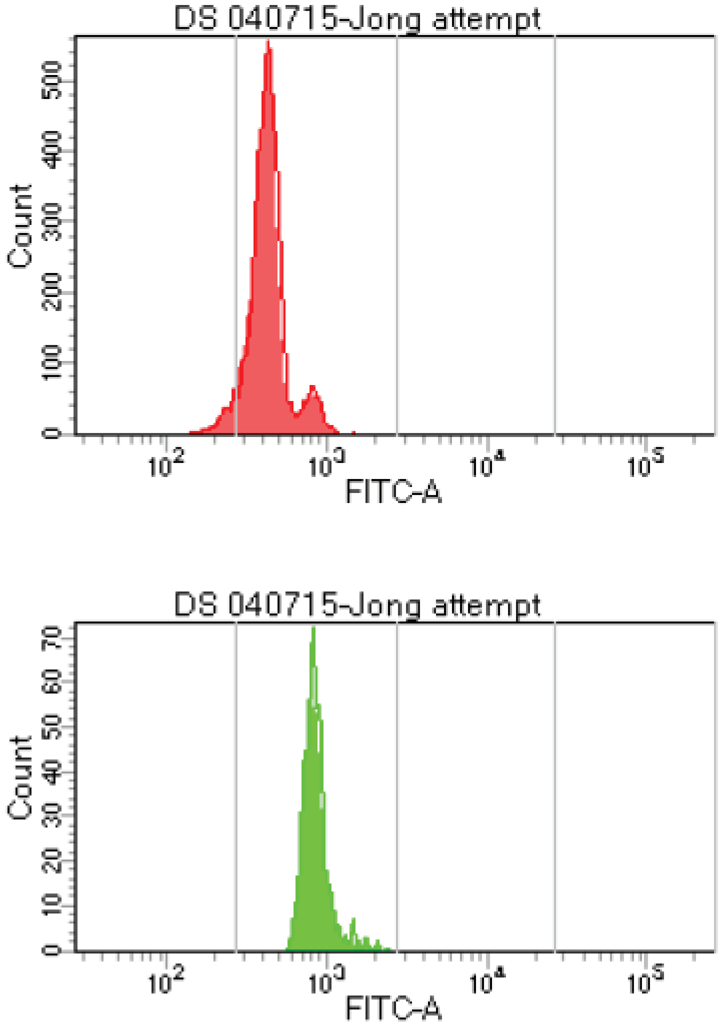

Figure S4. Flow cytometry/ FACS of the fractions of the agarose TXTL beads activated with HSL and fluorescing (A.) The total population sorted by flow cytometry and gated appropriately (B.) The activated population (green) differentiated by the non-activated population (red).

Figure S5

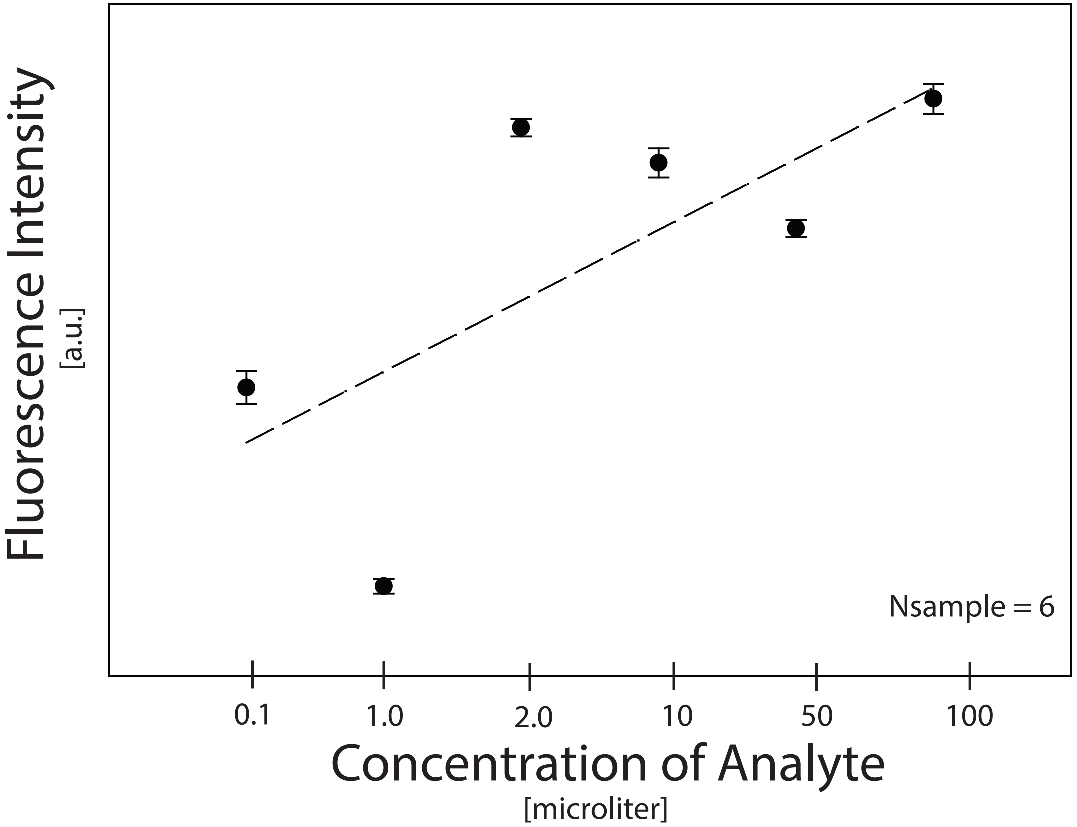

Figure S5. Plot of GFP signal intensity of the LasR-based agarose TXTL bead biosensor system in a real-world biological environment such as fecal analyte observed via plate-based fluorescence assay as a function of concentration of analyte

Figure S6

A. Comparative Protein Sequences

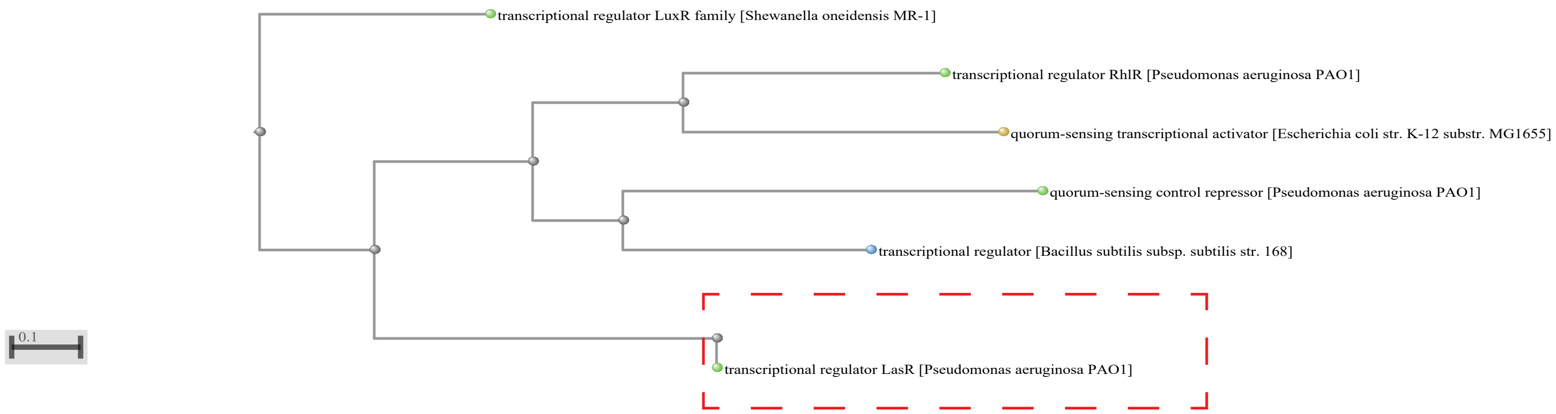

B. Consensus DNA-binding Sequences to LasR

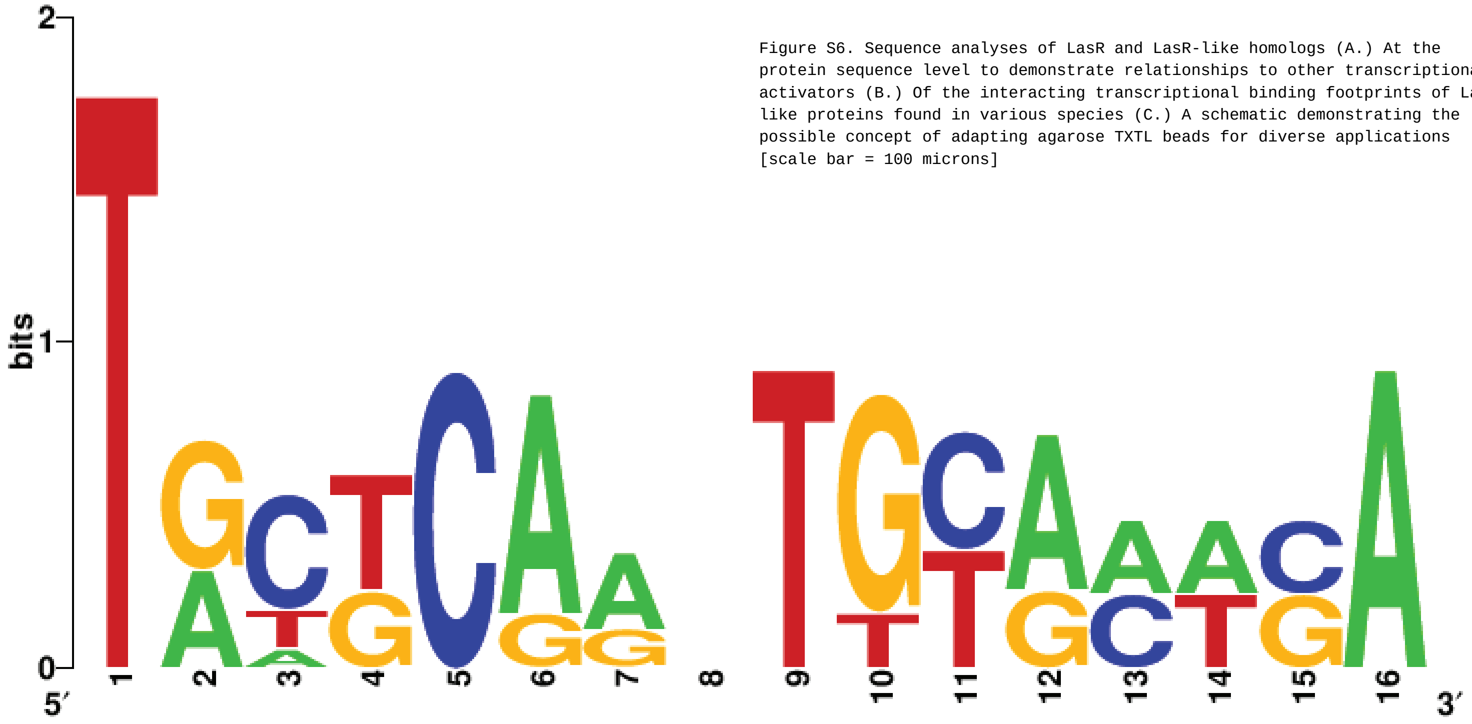

Figure S6. Sequence analyses of LasR and LasR-like homologs (A.) At the protein sequence level to demonstrate relationships to other transcriptional activators (B.) Of the interacting transcriptional binding footprints of LasR-like proteins found in various species (C.) A schematic demonstrating the possible concept of adapting agarose TXTL beads for diverse applications [scale bar = 100 microns]

C. Adapting agarose TXTL for various Applications

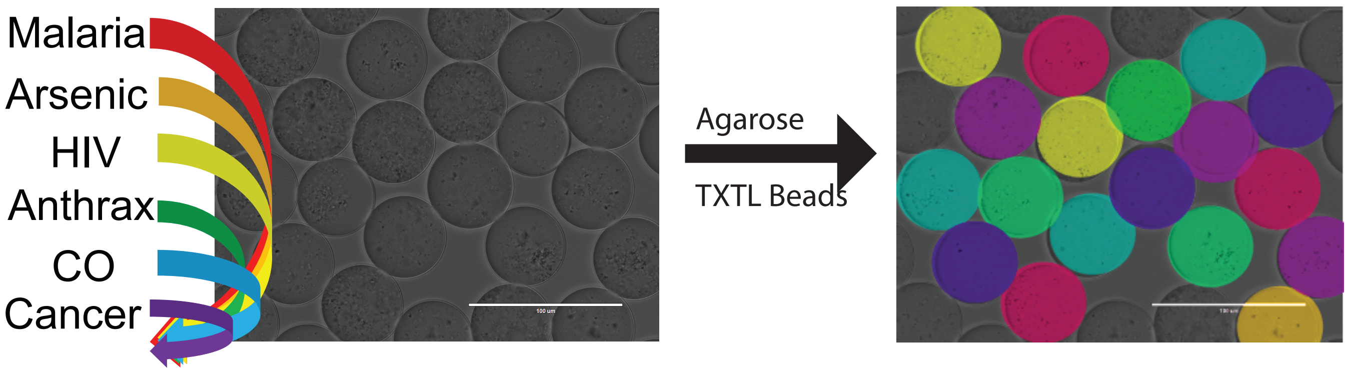

Movie S1. 3D SIM imaging of the agarose TXTL beads in culture with *Pseudomonas* cells. An animation of the 3D reconstruction of *Pseudomonas aeruginosa* and activated agarose TXTL beads
